# Implementing Advanced HIV Disease Care for Inpatients in a Referral Hospital in Malawi – Demand, Results and Cost Implications

**DOI:** 10.5334/aogh.3532

**Published:** 2022-03-08

**Authors:** Tom Heller, Douglas Damba, Tapiwa Kumwenda, Jacqueline Huwa, Christine Kamamia, Angellina Nhlema, Claudia Wallrauch, Chimwemwe Chawinga, Cecilia Kanyama, Lillian Gondwe-Chunda, Jonathan Ngoma, Beatrice Matanje, Hannock Tweya

**Affiliations:** 1 Lighthouse Trust, Lilongwe, Malawi; 2 International Training and Education Center for Health, University of Washington, Seattle, WA, United States; 3 UNC Project, Lilongwe, Malawi; 4 Department of Medicine, Kamuzu Central Hospital, Lilongwe, Malawi

## Abstract

**Setting:**

100 bed medical ward in referral hospital, Lilongwe, Malawi.

**Objective:**

HIV positive patients admitted to hospital often have advanced HIV disease (AHD) and are at risk for mortality. WHO guidelines suggest a package of care for AHD; these are often not implemented, especially in inpatient settings. We describe an implementation model for AHD care, its outcomes in routine care and provide cost estimates.

**Design:**

An “AHD care room” was established staffed by HIV counselor, nurse, and clinical officer allowing Provider Initiated Testing and Counseling, diagnostic testing for AHD and ensuring availability of HIV and TB drugs for rapid treatment initiation.

**Results:**

In the observation period from January to December 2020, a total of 1549 medical inpatients were tested for HIV (coverage 77.1%); 69 tested positive (yield 4.5%). The total proportion of HIV positive was 32.3% (638 already on ART and 69 newly diagnosed). CD4+ testing was done in 460 medical inpatients (65.1%); 245 (53.2%) were below 200 cells/ml and thus met definition of AHD. A total of 238 received S-CrAg tests; 39 (16.3%) were positive; 62 (28.3%) of 219 U-LAM tests were positive. The cost per identification of HIV positive patient was US$ 110.8; per AHD diagnosis between US$ 17.1 to 78.9; per positive S-CrAg test US$ 18.5 and per positive U-LAM test US$ 17.5.

**Conclusion:**

Our model successfully implemented AHD services according to WHO guidelines and provides basic costing data. Similar services could be implemented in other hospitals in LMICs.

## Introduction

While Malawi has reached the 90-90-90 UNAIDS goals in 2020 [1], HIV remains a burden on the country’s health system and economic development. Many patients are diagnosed HIV positive when already sick and require hospital admission. Others who recently started antiretroviral therapy (ART) are admitted due to opportunistic infections (OI) de-masked by immune-reconstitution inflammatory syndrome (IRIS). Patients who have been treated longer but on failing ART regimen may also present to hospitals with AHD. HIV patients admitted often have advanced HIV disease (AHD) and carry a high risk of mortality [2,3]. The most common cause of death of HIV positive inpatients in Low and middle-income countries (LMICs) identified through autopsies is tuberculosis (TB), which is often disseminated and is associated with progressive immunosuppression [4]. Other common causes include infections of the lung (e.g. bacterial and pneumocystis pneumonia) and of the central nervous system (e.g. cryptococcal meningitis [CM] or TB meningitis) [5]. The World Health Organization (WHO) published guidelines for the care of AHD in 2017 [6], but implementation requires significant resources. While many Malawian outpatient ART clinics are supported through external funding as part of the global HIV response, hospitals rarely receive additional funds while caring for the sickest segment of the HIV patient population.

To respond to this gap, we established a model of service delivery to support the care of HIV-positive adult patients admitted to referral hospitals in Malawi. In this article, we describe the focus areas of our model, analyze the demand and results in 2020, compare it to historical data before COVID-19 and attempt to estimate costs of components for management of HIV-infected inpatients.

## Study population, Design and Methods

### Setting

Malawi is a country in Sub-Saharan Africa with an HIV prevalence of 8.5% making good progress towards the UNAIDS 95-95-95 goals, with overall 92% of patients diagnosed, 95% of them on treatment and 94% of them virologically suppressed [1].

Kamuzu Central Hospital (KCH) is a tertiary hospital located in Lilongwe city, in the central region of Malawi. It has 750 beds for a catchment population of over 5 million. The Department of Medicine manages medical wards with approximately 100 beds. KCH is managed by the Ministry of Health (MoH) and resources are often limited. Lighthouse (LH), a Public Trust and WHO-recognized Center of Excellence for integrated HIV care, operates large patient-centered HIV clinics across the country. Within KCH campus, it provides free outpatient HIV care for more than 11 500 patients on Antiretroviral Therapy (ART). LH prioritizes integration of TB and HIV services, and all patients starting ART are assessed for AHD according to WHO guidelines. LH is partially funded by MoH but receives significant external funding from the United States President’s Emergency Plan for AIDS Relief (PEPFAR) through the Centers for Disease Control and Prevention (CDC).

### Intervention design

Upon discussion with KCH management, LH has formally supported AHD inpatient care since 2016. A dedicated “AHD room” for screening and management was set up in the medical ward, equipped with electronic medical record system for patient registration. The AHD room is staffed by an HIV counselor, a nurse, and a part time clinical officer (all from LH clinic; clinical officer approx. 25% time equivalent). The HIV/TB care model supports the following areas of care (summarized in ***Box 1***).

**Box 1 Lighthouse Advanced HIV disease (AHD) inpatient care package**

* Recommended as components of the package of care for people with advanced HIV disease, WHO Guidelines for Managing advanced HIV disease p.9, 2017.

Comprehensive PITC

Linkage to in-ward HIV care and antiretroviral therapy

Access to diagnostic tests for advanced HIV disease

- POC CD4 count for HIV positive patients
- Urine-LAM for patients with CD4 <200*
- Serum-CrAg screening for patients with CD4 <200*
- Support with lumbar puncture and CSF-CrAg
- Focused assessment with sonography for HIV/TB (“FASH ultrasound”)
- Sample collection for GeneXpert MTB/RIF (sputum and other body fluids) (test done in central lab)* and GeneXpert HIVquant (test done at Lighthouse)

Availability of ART and CPT drugs in ward for rapid initiation*

Availability of TB treatment or IPT* and pyridoxine in ward for rapid initiation

Availability of cryptococcal treatment

- Liposomal amphotericin B/flucytosine for CM
- Fluconazole pre-emptive treatment for cryptococcemia*

Support in KS diagnosis and treatment through assessment and referral to clinic

#### Comprehensive Provider Initiate Testing and Counseling (PITC)

One HIV counselor is assigned full time to medical wards; HIV status is routinely ascertained for all patients admitted from Monday through Saturday. Patients already on ART are counseled, and all other patients are considered eligible for (re-)testing unless there is documentation of a negative test result within the previous 6 weeks. HIV positive patients are linked to HIV care to the AHD room.

#### Diagnostic testing for advanced HIV disease

Point-of-care (POC) CD4 tests are provided for all newly diagnosed HIV patients and upon request also for patients already on ART. The nurse performs CD4 test using rapid POC-technology (PIMA, Abbott, IL). Serum cryptococcal antigen (S-S-, IMMY, OK) to diagnose cryptococcemia and urine-LAM testing (Determine TB, Abbott, IL) to assess for TB cell wall components in urine which are suggesting disseminated TB is routinely done for all patients with a CD4 count below 200 cells/mm^3^. CD4 count, S-CrAg and U-LAM results are reported back to the treating team, and the LH clinical officer is immediately informed about positive results to initiate further diagnostic work-up and/or treatment. Beyond these routine POC-tests other diagnostic modalities supported by LH team include (1) support for sample and result logistic for GeneXpert MTB/RIF (Cephid, CA) performed in the main hospital laboratory for sputum samples, (2) support chest X-rays logistic to detect pulmonary TB available through the hospital radiology department; (3) provision of bed-site Focused Assessment with Sonography for HIV/TB (FASH) ultrasound to detect pleural and pericardial effusions, enlarged lymph nodes or splenic micro-abscesses as signs of extra-pulmonary tuberculosis (EPTB) by trained LH clinical officers [7] using a portable ultrasound machine, (4) POC-Viral Load (GeneXpert HIVquant, Cepheid) for patients with suspected ART failure offered at Lighthouse [8].

#### Availability of HIV and TB drugs in the ward for rapid treatment initiation

Except for patients with intracranial infections, HIV positive patients are eligible for initiating ART immediately according to Malawi’s “test and treat” approach [9]. Standard ARVs and TB medication are stocked in the AHD room to ensure immediate initiation of ART and TB treatment after diagnosis; TB treatment is supplied to HIV positive and negative patients on the day of TB diagnosis; Isoniazid preventive therapy as well as pyridoxine is available. Diagnostic lumbar punctures are done in all patients who test positive for S-CrAg and if CSF-CrAg is positive patients are treated with amphotericin B plus flucytosine provided. For patients with cryptococcemia and negative CSF-CrAg, preemptive oral fluconazole is provided. All drugs are supplied by the LH pharmacy directly in the ward, drugs dispensed are reconciled with the central stock register at LH. As some patients in 2020 were recruited in a CM treatment trial [10], study clinicians provided their treatment. Patients were included in the cost analysis assuming standard treatment and monitoring according to National guidelines [9].

### Data analysis methods

The number of patients tested in the medical wards as well as AHD tests performed for the medical ward were analyzed in detail. For the POC-tests and outcomes, as well as for patients initiating CM treatment, improvised paper registers are used. Additionally, patients initiating ART or TB treatment are recorded in standard MoH registers. These are part of the LH routine M&E program setup, and no additional data is collected. Comprehensive data was available for 2020 when the COVID pandemic caused widespread disruptions to health services and many patients avoided visiting facilities out of fear from COVID-19. A similarly complete data set was available from 2017, the first year of implementation of the inpatient services when POC services were only provided in the medical wards, this data is reported as historic “pre-COVID” level. The Malawi National Health Science Research Committee granted LH approval (NHSRC Protocol #829) for collection and use of clinical and programmatic data as used in this survey. To estimate the cost of the intervention, staff time was assessed using average 2020 salary costs; test consumables and equipment were valued according to current Global Fund and local pricing as applicable in Malawi (pers. comm. DHA).

## Results

### HIV testing for medical inpatients

A total of 3 340 patient admissions were recorded in medical wards in 2020 (***Table 1***). Among these, 390 patients 11.7% had died and 302 (9.0%) were discharged before being seen by the HIV counselor for example due to admission during weekends or overnight. A total of 638 (19.1% of total admissions) were already on ART. Of the remaining 2010 patients, 1549 were HIV tested (test coverage 77.1%) and 69 were identified HIV positive during this hospital stay (4.5% positivity among those tested); all newly positive patients were linked to the AHD room. The remaining patients were not tested as they had recent negative HIV tests documented or in rare cases (1.8% of all not tested) declined testing. Out of the 2187 patients (65.5% of all admissions) who had their HIV status ascertained during this hospital stay 32.3% (638 on ART plus 69 new) were HIV positive.

**Table 1:** HIV testing in medical wards and advanced HIV disease test data for KCH inpatients (January to December).


	2017	2020

**Admissions**	6645	3340

**Early death**	880 (13.2%)	390 (11.7%)

**Early discharge**	391 (5.9%)	302 (9.0%)

**On previous ART**	1614	638

**Eligible for testing**	3760	2010

**Tested**	3389 (90.1%)	1549 (77.1%)

**HIV positive**	240 (7.1%)	69 (4.5%)

**CD4 done**	583	460

**CD4 < 200**	n.a.	245 (53.3%)

**CD4 < 100**	230 (39.4%)	147 (31.9%)

**CrAg test done**	185	238

**CrAg positive**	20 (10.8%)	39 (16.4%)

**LAM tests done**	298	219

**LAM positive**	69 (23.1%)	62 (28.3%)


### Services for advanced HIV disease

Out of the total 707 HIV positive patients in the medical ward, 460 (65.1%) received CD4 testing (***Table 2***). Reasons for not testing were early discharge, death before testing and unavailability of test cartridges although the individual reasons were not documented. Of these 460 patients, 334 (72.6%) were already on ART and 126 (27.4%) were currently not on ART (69 new HIV positives and 57 who stopped ART). The duration of previous ART was not documented. Of the total CD4 tests done, 232 (50.4%) were done for female patients, 245 (53.3%) were below 200 cells/ml and therefore met the definition of AHD. The proportion of male patients with AHD was higher (61.0%) than in female patients (45.7%). A subset of 147 (32.0%) patients had CD4 counts below 100 cells/ml, with 37.3% of male and 26.7% of female patients with very low CD4 counts (difference not statistically significant).

**Table 2:** Characteristics of ART and TB patients newly starting treatment as inpatients in KCH wards (January to December).


	2017		2020	
	
	ART PATIENTS INITIATED	TB TREATMENT INITIALED	ART PATIENTS INITIATED	TB TREATMENT INITIALED

**Total**	274	306	117	201

**Male**	124 (45.2%)	173 (56.6%)	54 (46.2%)	116 (57.7%)

**Female**	150 (54.8%)	133 (43.4%)	63 (53.8%)	85 (42.3%)

**Age <20**	9 (3.2%)	15 (4.9%)	11 (9.4%)	9 (4.5%)

**20–50**	234 (85.5%)	250 (81.7%)	101 (86.3%)	151 (75.1%)

**>50**	31 (11.3%)	41 (13.4%)	5 (4.3%)	41 (20.4%)

**Medical ward**	204 (74.4%)	–	90 (76.9%)	–

**Other ward**	70 (25.6%)	–	27 (23.1%)	–

**WHO stage 1/2**	167 (60.9%)	–	81 (69.2%)	–

**WHO Stage 3/4**	107 (30.1%)	–	36 (30.8%)	–

**PTB only**	–	86 (28.1%)	–	26 (12.9%)

**EPTB (+/–PTB)**	–	220 (71.9%)	–	175 (87.1%)

**HIV positive**	–	243 (79.4%)	–	156 (77.6%)

**HIV negative**	–	63 (20.6%)	–	45 (22.4%)


A total of 238 (97.1%) AHD patients received S-CrAg tests and 37 (15.5%) were CrAg positive; 24 (64.9%) of these received a lumbar puncture and CrAg was positive in 15 (62.5%) CSF samples. There were 219 (89.4%) U-LAM tests performed of which 62 (28.3%) were positive (***Table 1***). In eight patients S-CrAg and U-LAM were both positive.

The proportion of HIV patients not on ART was similar among all patients tested for CD4 (27.4%) and the subgroups with CD4 count <200 cells (28.2%), with S-CrAg positive (24.3%), CSF-CrAg positive (31.2%) or U-LAM positive (29.0%). As the “AHD room” supports also other wards (gynecology, pediatric, A&E) in the hospital, an additional 73 CD4 tests (30 results <200 cells/ml), 33 CrAg test (2 positive) and 43 LAM tests (7 positive) were performed for these wards the patients were not included in the analysis of medical inpatients above but were included in the cost analysis below.

Servicing all wards in hospital, a total of 117 patients started ART through the “AHD room”, 46.2% were male and 30.8% were classified as WHO stage 3 or 4. Among the 201 patients who started TB treatment (male 57.7%), the majority (77.6%) were HIV positive; 87.1% were recorded to have EPTB. Fifty-nine patients were treated for Cryptococcal meningitis (CM) receiving liposomal amphotericin B and flucytosine in the observation period, many of them referred from other institutions.

### Focuses Assessment with Sonography for HIV-associated TB (FASH)

One hundred and forty (57.1%) of the 245 AHD patients received FASH ultrasound as it requires specialized clinical officer who were sometimes not available. In 47 patients (33.6%) the scan was recorded “positive”; Specific FASH findings were not documented. In the 134 AHD patients for whom FASH scan and U-LAM test were done, there was a diagnostic concordance of (63.3%), (Cohen’ k = 0.15 suggesting slight agreement); a number of patients were only positive in either test (***Table 3***). FASH contributed an additional 28 TB diagnoses, that were detected with U-LAM; the patients initiated TB treatment as indicated.

**Table 3:** Correlation of Urine-LAM and FASH ultrasound results in medical inpatients receiving both tests.


	FASH +	FASH –	

**U-LAM+**	17 (12.6%)	21 (15.7%)	38

**U-LAM–**	28 (20.9%)	68 (50.7%)	96

	45	89	134


% of agreement: 63.23%, Cohen’s k: 0.15 (Slight agreement).

### Comparison with AHD inpatient service provision in 2017

The total number of admissions in medical wards was almost twice as high in 2017 compared with 2020 (***Table 1***). The proportion of early deaths, patients already on ART and AHD screening coverage was similar in both years. Positivity among patients tested for HIV was significantly higher in 2017 (7.1% vs. 4.5%, p < 0.05). The number of CD4 tests done in 2017 was 583, in this period a threshold of 100 cells/ml were used to trigger S-CrAg and U-LAM tests. The number of these tests and the positivity rate are shown in ***Table 1***; the number of patients receiving ART and TB treatment in 2017 is shown in ***Table 2***.

### Estimated costs of AHD services in 2020

The full implementation cost is summarized in ***Table 4***. Capital investments included establishment of the AHD room and equipment, which amounted US$ 10 708. Given that the room has been in use for 5 years, the annual capital investment was assumed as 20% (US$ 2 141) of the initial set-up cost. Operational costs were grouped into salaries, test kits, drugs and an amount for other consumables. The annual average operational cost was US$ 55 173, or US$ 4 598 per month. Routine HIV status ascertainment accounted for 13.8% of the total annual cost, driven by the salary of the full-time HIV counselor and the expenses for the HIV tests. The average cost for one HIV test was US$ 4.9, and for the identification of one HIV positive patient US$ 110.8 (***Table 5***).

**Table 4:** Cost analysis of the Lighthouse inpatient AHD support program 2020.


	N	ITEM COST (USD)	

ROOM AND EQUIPMENT (CAPITAL INVESTMENT)			TOTAL/5 YEAR PERIOD

Room			in kind Dept. of Medicine

Face lifting and security upgrade (burglar bars, new locks etc.)	1	400	$400

EMRS screen, label printer	1	1 063	$1 063

PIMA CD4 count analyzer	1	8 995	$8 995

Other items (furniture, BP machine, scale, fan etc.)	1	250	$250

**Total**			$10 708

***Annual subtotal***			**$2 142/year**

**STAFF SALARY**			**TOTAL/YEAR**

LH HIV counselor (per year)	1	5 800	$5 800#

LH nurse (per year)	1	17 000	$17 000*

LH clinical officer (per year)	0.25	18 800	$4 700

***Subtotal***			**$27 500/year**

**TESTS**			

HIV rapid tests	1549	1	$1 549#

HIV confirmation tests	150	2	$300#

CD4 tests	533	8.4	$4 472*

CD4 PIMA beads for calibration	2	59.1	$118*

CrAg tests	271	2.8	$759**

LAM tests	262	4.6	$1 205***

EDTA tubes and needles	600	0.2	$120*

Creatinine, potassium and FBC monitoring (twice per CM treatment)	118	5	$590^&^

***Subtotal***			**$9 113/year**

**DRUGS**			

ART and CPT			part of long-term care (through Dept. HIV/AIDS)

RHZE and Vit B6			part of long-term care (through Nat. TB program)

Fluconazole			part of long-term care (through Dept. HIV/AIDS)

Liposomal AmphothericinB (per course)	59	186.9	$11 027^&^

Flucytosine (per course)	59	86.2	$5 086^&^

***Subtotal***			**$16 113/year**

**OTHER CONSUMABLES**			

Registers, stationary etc. per month	12	25	$300

**Total**			**$62 139/year (= $5.178/month)**


^#^ Cost of HIV testing, * cost of CD4 testing, ** cost of CrAg, *** cost of LAM, ^&^ cost of CM treatment.

**Table 5:** Cost of individual test and results obtained.


TEST AND RESULT TYPE	TOTAL COST	N	COST/CLIENT

HIV test done	US$ 7 649	1,549	US$ 4.9

HIV positive patient identified	US$ 7 649	69	US$ 110.8

CD4 test done (nurse time fully excluded)	US$ 4 715	533	US$ 8.8

CD4 test done (nurse time fully included)	US$ 21 715	533	US$ 40.7

CD4 < 200 identified (nurse time fully excluded)	US$ 4 715	275	US$ 17.1

CD4 < 200 identified (nurse time fully included)	US$ 21 715	275	US$ 78.9

Positive serum CrAg result	US$ 759	41	US$ 18.5

Positive urine LAM result	US$ 1 205	69	US$ 17.5

CM Patient treated w AmpB/FluC	US$ 16 703	59	US$ 283.1


When the nurse’s salary was fully included in the costs for CD4 testing, the cost was US$ 40.7 per CD4 test done and US$ 78.9 was spent per AHD diagnosis (CD4 <200). Excluding the nurse salary, the cost per test reduced to US$ 8.8 and US$ 17.1 per AHD diagnosis. Furthermore, while still excluding the nurse’s salary, the test costs per positive S-CrAg test were US$ 18.5 and per positive LAM test US$ 17.5 after CD4 screening. The cost of treating one CM patient with amphotericin B plus flucytosine was US$ 283.1 (cost of drugs plus lab monitoring, excluding nurse time or routine intravenous items and fluconazole).

## Discussion

Our service model provided successful implementation of AHD care model in the medical department of a tertiary-level governmental referral hospital. The facility cares for a very sick patient population, exemplified by the proportion of patients who die before they can even be approached for HIV testing. For patients reached, the testing coverage of 81.1% achieved in our model can be considered high for a routine service; ascertainment of HIV status is an essential test in this patient population and can be compared to a “vital sign” [11]. The availability of ART services in the ward allowed same-day ART initiation to be offered to all HIV patients following confirmed HIV diagnosis and assessment of AHD and cerebral infections as recommended [6]. Beside the early initiation, the provision of emergency ART proved also useful to prevent treatment interruptions due to missing drugs in the wards.

Medical inpatients are generally expected to have higher HIV prevalence than general populations, as they are sicker. In our data with overall prevalence of 32.3% HIV positive medical inpatients in 2020 reflects this considering an estimated national HIV prevalence of 8.5% [1]. The proportion of 4.5% HIV positive among those tested seems low, especially considering the drop from 7.1% in 2017. This is most likely due to Malawi’s active HIV program due to which the number of un-identified HIV positives is decreasing. Recent studies have shown that ART scale-up in Malawi has significantly reduced HIV positive hospital admissions [12]. On a national level the yield of HIV tests has also decreased and is found currently in 2020 at 3.0% (Department of HIV/AIDS, pers. com.).

Of the medical inpatients tested in our cohort, 53.3% had AHD as defined by a CD4 count below 200 cells/mm^3^, 32.0% even below 100 cells/ml, underlining the importance of AHD care in our patient population. As expected, this percentage is higher than reported from outpatient populations, in South Africa nationally 10.1% were found to have CD4 cells <100 cells/ml [13]. Still, it is lower than those reported in earlier cohorts of inpatients from DR Congo (2015) and Kenya (2017) were rated above 80% were observed [14]. Of the patients with CD4, 72.7% tests were already on ART at the time of admission, a similar proportion was seen among those with a low CD4 count. From our data we cannot discriminate whether these patients have already been ill when recently initiated and then continued deteriorating to require admission, or whether they were stable on treatment and developed adherence and/or resistance problems leading to failure of treatment and immunological failure. Nevertheless, it underlines the importance for not restricting AHD care to newly initiating patients. Patients “recently started” and those failing ART share substantial risk as was recently also reported from other settings [14] and therefore need to be tested.

Screening coverage of S-CrAg and U-LAM among those with low CD4 (below 100 cells in 2017, below 200 cells in 2020) was very high (>95%) due to POC reflex testing provided by the nurse immediately. S-CrAg screening was positive in 16.4% of the medical patients tested. Recent data from South Africa reported %CrAg positivity in samples with CD4 counts <100 cells/ml of 5.4% nationally, in some districts in KwaZulu-Natal even up to 8–10% [13].

Access to lumbar puncture, in S-CrAg positive patients was 64.9%, mostly done by a clinical officer, and revealed CM in more than half of the cases stressing the need to perform a lumbar puncture in all S-CrAg positive inpatients. CM is treated with liposomal amphotericin B and flucytosine, those with cryptococcemia initiated pre-emptive treatment with fluconazole as recommended by WHO [15]. The high U-LAM positivity in our population is consistent with reported high positivity in patients with low CD4 counts [16], especially in those patients who are unable to provide sputum samples [17]. Nevertheless, the high proportion also may raise the question of potentially false-positive test readings.

In our patients, the search for sonographic findings suggestive of extra-pulmonary TB like pericardial effusions, abdominal lymphadenopathy and splenic micro abscesses as described in the FASH protocol [18] showed an overlap with positive U-LAM tests, but contributed and additional 28 TB diagnoses where U-LAM was negative. This may have included patients with pleural or pericardial TB which may often be LAM negative. Nevertheless, the generally large proportion of inpatients treated for TB being HIV positive, the high rate of positive U-LAM tests and of FASH scans as well as the high proportion of EPTB reported may in some suggest that disseminated TB was present in a large proportion of inpatients as previously observed [4].

When comparing our data to historical data from 2017, the most impressive change is the reduction of total admissions, which halved in 2020. This is likely due to COVID-19 as the years 2018 and 2019 showed similar overall numbers of medical admissions at Kamuzu Central hospital (pers. comm., KCH administration). Patient’s difficulties in reaching healthcare facilities due to lockdown but also fear of COVID-19 discouraged healthcare seeking [19]. This would explain the lower number of admissions and in consequence lower number of HIV diagnosis and HIV and TB cases treated. The proportions of patients with AHD and with positive S-CrAg and U-LAM tests were not very different between the two observational periods. How much COVID-19 will overall impact on the HIV and TB epidemics remains to be seen in the future [20].

The “fully loaded” cost per HIV test performed health facilities in Malawi has been previously reported at US$ 4.9, which is similar to US$ 4.2 in neighboring Zambia but lower than the US$ 8.8 in Zimbabwe. The same study estimated mean cost per HIV-positive individual identified at US$ 79.6 in Malawi (Zambia: US$ 73.6, Zimbabwe US$ 178.9) [21]. More recent data from Malawi suggested the average cost per testing episode at US$ 2.8 and average cost per HIV diagnosis at US$ 116.3. As staff salaries contribute substantially to costs, highest costs found in facilities with the lowest daily number of tests and lowest HIV yield respectively [22]. Our data shows US$ 4.9 per HIV test and US$ 110.8 per HIV positive patient identified, this is well in line with the reported data and suggests that our costing assumptions are realistic.

Few studies have explored costs associated with the care of AHD. A study from Tanzania reported costs for CD4 counts (US$ 15.8) and S-CrAg (US$ 8.4). The study did not report urine-LAM costs, nor did it include nurses’ time or provides sufficient detail to allow cost estimates per positive tests [23]. Urine-based tuberculosis screening for hospitalized patients with HIV was found cost-effective in modeling studies for resource-limited settings [24], the actual costs for implementation were not assessed or reported in this report. In our study, the costs per CD4 tests are estimated between US$ 8.8 and US$ 40.7, the cost per patient identified with CD4 <200 cells/ml between US$ 17.1 and US$ 78.9. The costs depend on the extent of nurse’s time that is included in the cost; to estimate the range we calculated the lower margin, excluding it and the upper margin including it fully. The true cost can be assumed in between, as the nurse spends time performing CD4 tests but also spends large parts of her time on other duties like ART counseling, S-CrAg and U-LAM testing or administration of ART, TB or CM drugs. In patients identified with AHD, we estimate costs per positive S-CrAg test at US$ 18.5 and per positive U-LAM test at US$ 17.5; these costs are largely dependent on prevalence of both conditions among inpatients. The costs of treating an episode of CM with amphotericin B plus flucytosine were estimated US$ 283.1, which is relatively high and mainly driven by the drug prices. However, the relatively small number of CM cases diagnosed and treated, as well as the lifesaving role of appropriate treatment make cost considerations for antifungal drugs less relevant.

It is an interesting anomaly in our cost analysis, that the more expensive CD4 test, which additionally requires electronic equipment, is performed to identify patients eligible for the comparatively cheaper tests of S-CrAg and U-LAM. To our knowledge no studies exist examining the cost and efficacy of other possible testing algorithms. Our algorithm follows WHO guidance and CD4 counts are helpful beyond defining eligibility of patients for AHD screening, e.g. to assess the probability of pneumocystis pneumonia in patients with shortness of breath or of toxoplasmosis with focal neurological symptoms. However, unconditional screening with U-LAM and S-CrAg in this patient population, and only targeted use of CD4 might be cost-saving and facilitate implementation.

One limitation of our study is that our results are based on routine data, as such are less accurate and less complete than data from well-controlled clinical studies. Nevertheless, we feel that the data gives realistic picture of the efforts and possible achievements on the ground. The cost estimates are approximate and depend critically on the prevalence of TB and CM, which may differ widely in other settings. Therefore, the generalizability of our referral hospital results to district hospitals can be debated. With fixed costs for equipment and staffing, but smaller patient numbers in district hospitals, the cost-effectiveness analysis may yield different results. In any case, it can be stated that significant incremental costs need to be covered to allow WHO AHD guideline implementation. As many hospitals in LMICs struggle financially and are often underfunded, these needs are likely to require support by external funding.

We feel that our model has successfully implemented following WHO AHD guidelines for sick in-patient populations. We have produced basic, but reasonable costing data that implies affordability and can be budgeted for with monitorable results. We think this model could be scaled-up and LH is planning to implement similar services in other hospitals.
